# Restoration of Range of Motion in the Cervical Spine through Single-Segment Artificial Disc Replacement Using the Baguera^®^C Prosthesis

**DOI:** 10.3390/jcm13072048

**Published:** 2024-04-02

**Authors:** Ming-Cheng Tsai, Ya-Fang Liu, Wei-Hsing Lin, Ming-Chung Lee

**Affiliations:** 1 School of Medicine, Fu Jen Catholic University, New Taipei City 242, Taiwan; 2 Neurosurgical Department, Shin-Kong Wu Ho-Su Memorial Hospital, Taipei 111, Taiwan; 3 Research Department, Shin-Kong Wu Ho-Su Memorial Hospital, Taipei 111, Taiwan; 4 Department of Life Science, National Taiwan Normal University, Taipei 116, Taiwan

**Keywords:** cervical spine motility, cervical disc arthroplasty (CDA), range of motion (ROM), ROM of C2–C7, ROM of functional spinal unit (FSU)

## Abstract

**Background:** Anterior cervical discectomy and fusion (ACDF) is a standard procedure for degenerative diseases of the cervical spine, providing nerve decompression and spinal stabilization. However, it limits cervical spine motility, restricts fused segment activity, and may lead to adjacent degeneration. Cervical disc arthroplasty (CDA) is an accepted alternative that preserves the structure and flexibility of the cervical spine. This study aimed to explore the dynamic changes in the range of motion (ROM) of the cervical spine after CDA using a viscoelastic artificial disc, as well as the factors affecting mobility restoration. **Methods:** A retrospective analysis was conducted on 132 patients who underwent single-level anterior cervical discectomy and CDA from January 2015 to June 2022. **Result:** Analysis of data from 132 patients revealed a significant improvement in clinical outcomes. The mean ROM of C2–C7 and functional spinal unit (FSU) segments significantly increased from 2 to 36 months post-operatively. Cervical spine flexibility was preserved and enhanced after prosthesis implantation. However, it took six months for the cervical spine motility to stabilize. In addition, sex and age were found to impact motility restoration, with female and younger patients exhibiting larger ROMs post-surgery. Additionally, CDA at the C5–C6 level resulted in the greatest increase in ROM, potentially improving overall kinematic ability. **Conclusions:** Single-segment artificial disc arthroplasty effectively restores the ROM in degenerative cervical spine conditions.

## 1. Introduction

The cervical spine in humans serves not only to support the weight of the head but also to facilitate multi-directional activities. As we age, the flexibility of the cervical spine decreases, which may lead to degenerative diseases [1]. These conditions, such as disc herniation, narrowed foramina, osteophyte formation, and endplate sclerosis, can potentially cause cervical myelopathy or radiculopathy [2]. Among these, cervical disc herniation is the most common degenerative cervical disease, particularly affecting individuals over 40 years of age [3].

Surgical intervention becomes an option for patients whose symptoms and neurological deficits cannot be alleviated by non-invasive or minimally invasive treatments [4,5]. Anterior cervical discectomy and fusion (ACDF) has been widely adopted as the gold standard treatment for cervical disc herniation and was first described in 1958 by Smith and Robinson. However, the fusion-based reconstructive procedures used in ACDF sacrifice the motility of the cervical spine and may result in future adjacent degenerative diseases. Therefore, numerous dynamic cervical implants have been developed since 2002, with several studies highlighting the benefits and complications of these devices [6,7].

Cervical disc arthroplasty (CDA) has gained acceptance as a reliable surgical alternative over the past decade [8]. The superiority of CDA over ACDF has been demonstrated by randomized controlled trials, with advantages including higher long-term clinical success rates, improved motion flexibility, reduced incidence of adjacent syndrome, lower reoperation rates, and shorter hospital stays [9,10,11,12]. In a recent systematic review evaluating the cost effectiveness of CDA and ACDF, the findings suggested that CDA may be a more cost-effective treatment approach [13].

The existing literature highlights the benefit of CDA in restoring the physiological biomechanical properties of the cervical spine [14,15,16]. Range of motion (ROM), which reflects cervical flexibility, is a well-studied biomechanical parameter for evaluating motion preservation. However, there is still a lack of comprehensive understanding regarding the dynamic changes in ROM after CDA with a viscoelastic artificial disc, particularly those that provide motion in 6 degrees of freedom (6-DOF). These new-generation prostheses are expected to resemble natural discs and hold promise for future CDA procedures [17].

The present study was designed as a three-year follow-up to investigate the mobility of the cervical spine after arthroplasty with a semi-constrained cervical disc prosthesis (Baguera^®^C, Spineart Geneva SA, Geneva, Switzerland). By analyzing the changes in ROM, which was measured by radiographic outcomes, this retrospective study aimed to elucidate whether age, sex, and operative level had an impact on mobility restoration after CDA with a viscoelastic disc.

## 2. Materials and Methods

### 2.1. Study Design and Patient Population

This study conducted a retrospective analysis of patients who underwent single-level anterior cervical discectomy and cervical disc arthroplasty (CDA) from January 2015 to June 2022 at our institute. All patients had cervical degenerative diseases with radiculopathy and/or myelopathy and underwent surgeries by the same surgeon. A total of 132 patients (60 males and 72 females) were enrolled in the study. The ages of the patients ranged from 32 to 88 years, with a mean age of 58.03 years at the time of surgery. The minimum follow-up period was six months.

The inclusion criteria specified that patients must have symptomatic cervical disc disease at a single level between C3–C4 and C6–C7. Conservative treatments had failed for all patients with herniated discs or bony osteophytes, as observed in their magnetic resonance imaging studies. The exclusion criteria comprised osteoporosis, rheumatoid arthritis or other autoimmune diseases, ossification of the posterior longitudinal ligament, active infection, structural instability, disc space collapse, known allergy to titanium, neoplasia, and metabolic bone diseases. All patients included in this study provided informed consent by accepting and signing a consent form. This study was approved by the Institutional Review Board of Shin Kong Wu Ho-Su Memorial Hospital (approval code: 20200202R) before data collection and analysis.

### 2.2. Device Description

All patients underwent CDA using the Baguera^®^C prosthesis (Spineart Inc., Plan-Les-Ouates, Switzerland), which enables motion in 6-DOF. The Baguera^®^C is a semi-constrained cervical disc prosthesis consisting of a high-density polyethylene nucleus that serves as an articulation between two titanium endplate components. It has a porous coated exterior and a diamond-like carbon-coated interior, as well as three fins on each endplate, providing immediate stability after implantation.

### 2.3. Surgical Procedures

The surgical procedure involved a modified Smith-Robinson anterior approach to the cervical spine. The same neurosurgeon performed the procedures for all 132 of the patients. The patient was placed in a supine position on the operating table with a soft pad placed under the shoulder to slightly extend the neck. A transverse skin incision of 2.5 cm was made on either the right or left side of the neck, depending on the laterality of the patient’s symptoms. Through this incision, the target level was reached and confirmed using intra-operative lateral fluoroscopy. A self-retractor was used to push the trachea and esophagus across the midline to the opposite direction and the carotid sheath laterally, exposing the anterior border of the cervical spine.

Discectomy was performed, removing all cartilaginous endplates, hard spurs, and the disc itself. The posterior longitudinal ligaments were opened in all cases to ensure sufficient decompression. Bilateral uncovertebral joints (Luschka’s joints) were also released. A properly sized Baguera^®^C cervical prosthesis was then implanted into the intervertebral space, guided by lateral fluoroscopy. An implant with dimensions close to the original disc space was selected. Any bone dust was irrigated out with regular saline, and bone edges were sealed with bone wax. The wound was closed in layers, and no drainage tube was left. Patients were able to move independently immediately after recovering from anesthesia. The use of a cervical collar post-operatively was not necessary. Patients were allowed to attempt oral intake on the same day. After a review by the medical team, they could be discharged on post-operative day 2 or 3.

### 2.4. Clinical Evaluation

All patients diagnosed with single-level cervical herniated disc disease underwent a comprehensive neurological examination and completed an extensive questionnaire that included the Visual Analogue Scale (VAS) to assess pain in the neck and limbs, the Neck Disability Index (NDI) to evaluate disability, and the Japanese Orthopedic Association (JOA) score. These assessments were conducted both pre-operatively and on post-operative day 2 or 3.

### 2.5. Radiological Evaluation

To evaluate the ROM of the cervical spine, dynamic studies were conducted to assess its motility. The ROM of the C2–C7 segment represents the overall motility of the entire cervical spine, while the ROM of the functional spinal unit (FSU), a combination of two adjacent vertebrae and the intervertebral disc, represents the motility specific to the CDA procedure.

**Figure 1:**
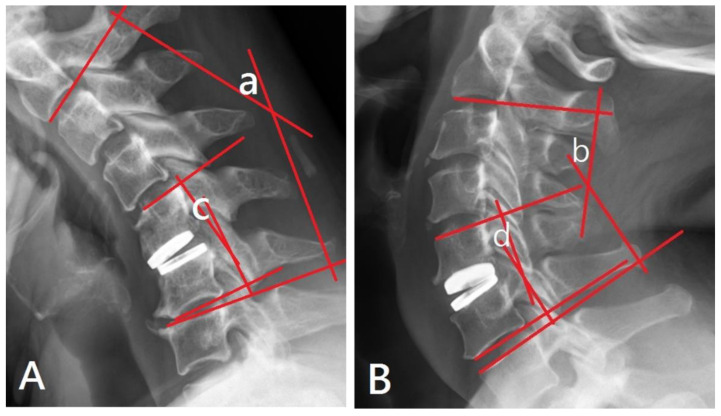
(**A**) (flexion view) and 1 (**B**) (extension view): Evaluation of the ROM of C2–C7 and FSU using flexion and extension lateral cervical radiographs. The angle of “a+b” represents the ROM of C2–C7. The angle of “c+d” represents the ROM of FSU.

The angles between the superior endplate of the upper vertebra and the inferior endplate of the lower vertebra were measured using Cobb’s technique on flexion and extension lateral cervical radiographs [18]. The sum of these two angles provided the ROM of the measured vertebrae. The ROM of C2–C7 extended from the inferior endplate of C2 to the superior endplate of C7, while the ROM of the FSU spanned from the superior endplate of the upper vertebra to the inferior endplate of the lower vertebra at the level of the lesioned disc (Figure 1).

Cervical spine radiographs in the anterior–posterior and lateral projections were taken on post-operative day 1 to assess the location of the implant. Dynamic studies, consisting of flexion and extension views in lateral projection radiographs, were performed at specific intervals (1 week, 1 month, 2 months, 3 months, 6 months, 12 months, 18 months, 24 months, and 36 months) to evaluate the motility of the cervical spine. To ensure consistency and minimize observer bias, two independent technicians evaluated the radiographs and calculated the angles to determine the ROM.

### 2.6. Radiological Evaluation

Statistical analyses were performed using SPSS version 26 (SPSS Inc., Chicago, IL, USA). The data were presented as mean ± SD. Comparisons between the two groups were performed using the Mann–Whitney U test, paired *t*-test, or Chi-square test. One-way analysis of variance (ANOVA) was used to compare the ROMs during the pre-operation period and the follow-up periods. To evaluate the effects of two different factors and their interaction, the general linear model (GLM) univariate analysis was employed. *p* values were set at *p* ≤ 0.05 to determine statistical significance.

## 3. Results

A total of 132 patients (60 females and 72 males) who underwent single-level surgery between January 2015 and June 2022 were included in this study. The surgical levels at which the patients underwent the procedure were C3–C4 (*n* = 25), C4–C5 (*n* = 15), C5–C6 (*n* = 50), and C6–C7 (*n* = 42). No significant differences between sexes were found at baseline (Table 1) with respect to age, mean pre-operative VAS scores, and ROM of the FSU and overall cervical spine (C2–C7).

### 3.1. Clinical Outcomes

Cervical disc arthroplasty surgery resulted in significant improvements in clinical outcomes, as evidenced by decreased VAS scores and NDI, and increased JOA scores (Table 2).

### 3.2. Radiological Evaluation

Radiological evaluations were conducted on each patient before surgery and at regular post-operative intervals (1 week, 1 month, 2 months, 3 months, 6 months, 12 months, 18 months, 24 months, and 36 months) for a period of three years. All patients completed the radiological evaluations, resulting in a follow-up rate of 100%.

#### 3.2.1. Improvement in ROM

The ROM showed significant improvement, indicating enhanced mobility of the cervical spine. Prior to surgery, the mean ROM for C2–C7 was 40.17° ± 13.37°, and for the FSU, it was 12.41° ± 6.77°. At the 36-month follow-up, these values increased to 47.31° ± 13.18° and 15.58° ± 6.52°, respectively. Compared with the pre-operative period, statistically significant upregulation of ROM for the FSU and C2–C7 was observed post-operatively from the 2-month follow-up and continued throughout the 36-month follow-up period.

**Figure 2:**
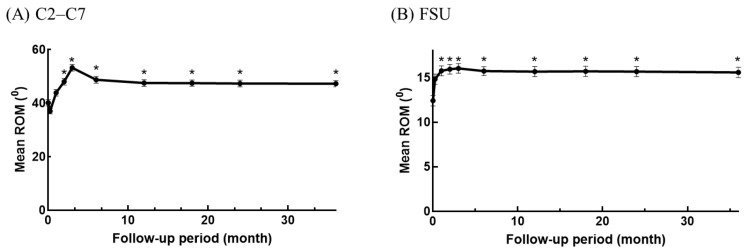
Dynamic changes in ROM of C2–C7 (**A**) and FSU (**B**). * *p* ≤ 0.05 compared to pre-operative baseline. The point on the y-axis is the ROM of the pre-operative period.

The highest mean ROM values were recorded at the 3-month follow-up (overall cervical spine: 53.3°, FSU: 16.02°). The ROM of the FSU remained well-maintained with less than 1° difference at any point from the 3-month throughout the 36-month follow-up. Although the mean ROM of the overall cervical spine slightly decreased at the 6-month follow-up (48.76°), the difference was not statistically significant compared to the 3-month follow-up. Subsequently, the ROM remained stable with a difference of less than 1.5° throughout the remaining follow-up period (Figure 2).

Compared to the pre-operative baseline measurements, the ROM of both C2–C7 and FSU increased significantly from the 2-month follow-up and continued throughout the 36-month follow-up. The radiological findings indicated that the ROM of both C2–C7 and FSU remained significantly improved from the 2-month follow-up to the last follow-up, suggesting sustained improvement over time. To further explore the factors influencing the improvement in cervical motility, longitudinal data were analyzed to examine the potential impact of age, sex, and the operated segment.

#### 3.2.2. Comparing the Improvement in ROM between Males and Females

**Figure 3:**
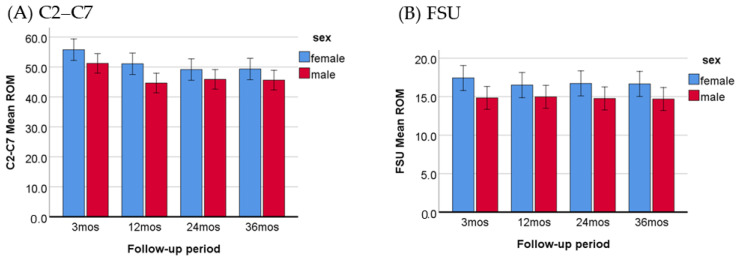
Comparison of C2–C7 (**A**) and FSU (**B**) ROM between females and males within 3–36 months post-operatively, with a *p*-value less than 0.001 for both ROM.

To evaluate potential differences in post-operative ROM between males and females, the ROM measurements at the 3-, 12-, 24-, and 36-month follow-ups were analyzed. As presented in Table 3 and Figure 3, the ROM of C2–C7 and FSU both increased significantly in females compared to males. Additionally, no interaction was observed between the time intervals and sex groups, indicating that the ROM improvements were consistent across the follow-up periods for both sexes.

#### 3.2.3. Comparing the Improvement in ROM between Older and Younger Patients

**Figure 4:**
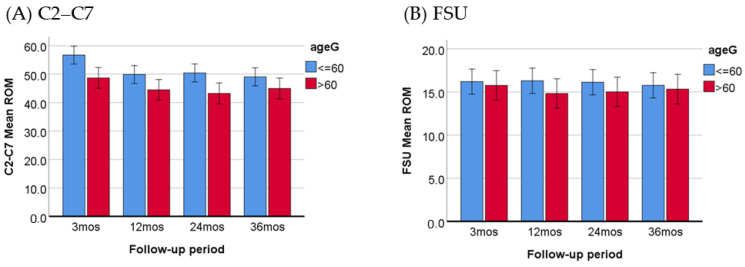
Comparison of C2–C7 (**A**) and FSU (**B**) ROM between younger patients and older patients within 3–36 months post-operatively, with a *p*-value less than 0.001 for C2–C7 ROM.

This study aimed to investigate whether age has an impact on the improvement in post-operative ROM. For the purpose of our analysis, we defined individuals aged 60 years or older as the “older adults” group, ensuring a relatively equal distribution of patients in both age groups (≤60, *n* = 76; >60, *n* = 56). The ROM measurements at the 3-, 12-, 24-, and 36-month follow-ups were analyzed. The mean pre-operative ROM of C2–C7 was 41.96 ± 13.73 (≤60 group) and 37.74 ± 12.57 (>60 group), showing no significant difference between the two groups (*p* = 0.072). However, there was a significant difference in the pre-operative ROM of the FSU, with values of 13.44 ± 7.49 (≤60 group) and 11.01 ± 5.41 (>60 group) (*p* = 0.042). Notably, the age difference was only observed in the ROM of C2–C7 after surgery, and there was no interaction between time and age groups (Table 4, Figure 4).

#### 3.2.4. Comparing the Improvement in ROM between Patients with Different Levels of Surgery

**Figure 5:**
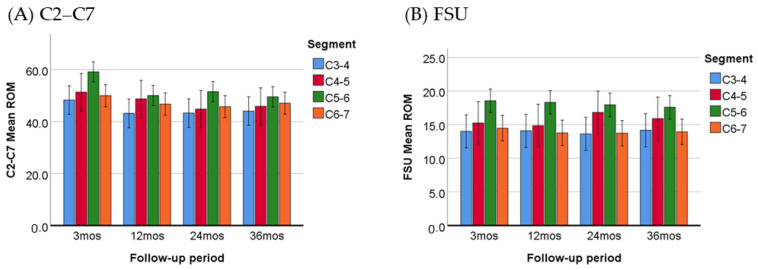
Comparison of C2–C7 (**A**) and FSU (**B**) ROM between patients treated on different segments. The increase in ROM was most pronounced in patients who were operated at the C5–C6 level.

The ROM was evaluated at various time intervals, including the 3-, 12-, 24-, and 36-month follow-ups. The results, presented in Table 5 and Figure 5, indicate that the increase in ROM was most pronounced in patients who were operated at the C5–C6 level. The ROM for both C2–C7 and FSU were significantly larger (*p* ≤ 0.05) than the other segments, with the exception of the ROM for C2–C7 at the C4–C5 operated level (*p* = 0.117).

In addition, heterotopic ossification (HO), which refers to the abnormal formation of bone tissue growing into the disc space and partially blocking the ROM, was found in 11 of the 132 patients (8.3%) at the final follow-up.

## 4. Discussion

The patient cohort examined in the current study demonstrated significant improvements in all clinical outcome parameters (NDI, VAS, and JOA) in comparison to baseline levels. Previous studies have also verified similar favorable clinical outcomes at different observation periods, irrespective of the type of prosthesis implanted [19,20,21,22]. It has been suggested that higher pre-operative NDI scores are associated with a more pronounced C2–C7 sagittal vertical axis and reduced cervical lordosis [23]. Following surgical reconstruction, which led to a decrease in NDI and VAS scores, CDA not only alleviates pain but also restores sagittal balance in the cervical spine.

A literature review has revealed that CDA preserves the motility of the cervical spine, especially at these operative levels [24,25,26]. However, there are inconsistent findings in clinical trials using different types of prostheses and varying follow-up periods regarding changes in ROM before and after surgery [27,28,29,30]. A study cohort using Baguera^®^C, which is the same type of prosthesis used in this study, reported a non-significant decrease in mean ROM of the FSU and a non-significant increase in ROM of C2–C7 two years after surgery [31]. A recent study on the longitudinal changes in ROM at the operated segments following CDA with the Mobi-C prosthesis, a semi-constrained artificial disc classified under the same category as the artificial disc used in our study, found a significant decrease in ROM starting 24 months after the operation [32].

In the present study, the mean ROM of C2–C7 and FSU increased at all follow-up points, with the exception of the ROM of C2–C7 at the first follow-up (one week after surgery). This temporary decrease was attributed to discomfort caused by the non-healing wound, which affected the patient’s ability to extend and flex their neck. All ROM upregulations were statistically significant in comparison to baseline levels from 2 months post-operatively to the final follow-up (Figure 1). The ROM remained relatively stable after the 6-month follow-up, indicating that the improved flexibility of the operated segment and the cervical spine was preserved well by the implanted viscoelastic prosthesis.

Additionally, this finding demonstrates that the stability of the motor function after intervertebral disc replacement requires a 6-month period of adaptation before reaching a stable plateau. Therefore, a 6-month follow-up is sufficient to assess the influence of CDA on cervical spine motility. The substantial improvement observed for three years after CDA, as well as the low ratio of HO in our cases, may be attributed to specific surgical techniques employed in this study. These techniques involved the decompression of bilateral Luschka’s joints (uncovertebral joints), thorough normal saline irrigation to remove bone dust, and the use of bone wax to seal bone edges after implantation.

Sex plays an influential role in determining the total ROM in the cervical spine of asymptomatic individuals, with females generally exhibiting significantly larger ROM compared to males [33,34]. In our patient cohort, no significant sex differences in ROM were observed at the baseline assessment (Table 1). However, the post-operative ROM of the total cervical spine and FSU increased significantly in females compared to males (Table 3, Figure 3). This finding suggests that the reconstruction of degenerative levels in female patients may lead to better cervical motility restoration, resulting in significantly larger ROM, which is in line with observations in the asymptomatic population. Thus, even after surgery, females are able to retain their innate advantages in cervical motility. In contrast to our study, Wu, T.K. et al. reported that sex and age did not have a significant effect on post-operative ROM [21]. Their study enrolled patients who underwent 1-level CDA (48.41%) and 2-level CDA (51.59%). The higher complexity of the study population may interfere with the analysis results.

The total cervical ROM tends to decrease with increasing age, primarily caused by the degeneration of the intervertebral discs [34,35]. In our study, the patients were divided into two groups: older (>60 years old) and younger patients (≤60 years old). Prior to surgery, there was no significant difference in the total cervical ROM between the two groups. This lack of difference may be due to neck pain or stiffness, which could limit the motility of the cervical spine. However, the post-operative ROM of the C2–C7 segment increased in both groups, but the values of ROM were significantly larger in the younger group (Table 4), indicating that the flexibility of the cervical spine was markedly improved by the implanted viscoelastic prosthesis in young individuals. Therefore, single-segment CDA appears to be particularly beneficial for younger patients.

It is noteworthy that the pre-operative ROM of the FSU at the operated level was significantly smaller in the older group, but this difference became non-significant after the implantation of the prosthesis (Table 4). A radiographic assessment performed on 212 asymptomatic volunteers found that age has a significant negative association with segmental ROM at all cervical levels, with segmental ROM decreasing at the degenerated disc levels [33]. The non-significant post-operative difference indicates that our patients, regardless of their age, were able to preserve segmental motility well after receiving the prosthesis replacement at the diseased segment. On the other hand, a comparative study found that younger patients (≤40 years old) had a significant post-operative segmental ROM than elderly patients (≥65) [36]. The reason for the disparity between this study’s findings and our own should be attributed to differences in the study cohort and prosthesis implanted.

Given the distinct differences in motion characteristics among different segments of the cervical spine during neck motion [37], it is expected that the outcomes of patients with implants at different levels would vary in terms of cervical motility. Although there was no significant difference in the pre-operative ROM of the C2–C7 segment among patients with different operative levels, the analysis of the longitudinal changes in ROM from three months to three years post-operatively revealed that the most significant increase in ROM occurred in patients treated at the C5–C6 level (Table 5, Figure 5). These patients demonstrated the greatest improvement in cervical spine flexibility post-operatively. Biomechanical and kinematic investigations have shown that the C5–C6 motion segment exhibits the maximum ROM for flexion/extension motion in healthy young adults [38]. Furthermore, a single-level degenerative condition at the C5–C6 segment leads to a substantial decrease of approximately 32% in flexion and 33% in extension in segmental ROM [15]. Moreover, in symptomatic patients with severely degenerated segments, the contribution of the C5–C6 motion segment to the total angular ROM decreases significantly [39]. Unfortunately, C5–C6 is one of the most commonly affected levels in degenerative disc disease. This indicates that degenerative cervical diseases affect the C5–C6 level frequently. After CDA at the diseased C5–C6 level, the function of the C5–C6 motion segment could be restored, and so could the contribution to ROM. It is rational to expect that patients treated at the C5–C6 level would have better post-operative ROM than others. According to our findings, patients who were operated at the C5–C6 level experienced the most significant improvement in ROM. The motility restoration in the C5–C6 motor segment appears to elevate the overall kinematic ability of the cervical spine more effectively than other segments.

While CDA is a more physiological surgical option for cervical degenerative diseases compared to ACDF, its high medical expenses limit its widespread adoption. For patients with multi-level cervical degenerative diseases, some surgeons have advocated for hybrid surgeries, which combine fusion and non-fusion procedures, as a cost-effective alternative for replacing diseased cervical discs [40]. Considering the pivotal role of the C5–C6 level in cervical spine motility, whether it is through single-segment CDA or hybrid surgeries, the replacement of the C5–C6 disc with an artificial disc proves to be the most cost-effective approach and should be given the highest priority in terms of restoring cervical spine ROM.

In addition, HO is a common post-operative complication in cervical spine arthroplasty, with reported incidences ranging from 5% to 63%. The occurrence of HO can vary depending on the type of prosthesis used and the length of the follow-up period [41,42]. HO may have a significant impact on the motility of the cervical spine after CDA, particularly in cases classified as McAfee’s class III and IV, where the ROM is severely restricted [41]. This suggests that the motility of the cervical spine may be preserved in cases classified as class 0 to II. Furthermore, it is challenging to evaluate the grading of HO accurately using plain radiographs alone. In our study, the occurrence of HO was approximately 8.3%. However, it is possible that this figure may be underestimated, and the incidence may increase with further long-term follow-up.

A survey estimating the incidence of cervical spine surgery from 2020 to 2040 in the United States predicts an increase in cervical spinal disease and a subsequent rise in surgical volumes in the near future [43]. Given the allowance for 6-DOF motion, viscoelastic devices have been recommended as ideal artificial discs that resemble the physiological motion patterns of natural discs [44]. The results of this study provide valuable insights into the changes in ROM that occur over a three-year period following CDA with a viscoelastic device. It highlights the significance of the C5–C6 segment for cervical spine ROM and suggests that a 6-month adaptation period is required to reach a stable ROM status. Single-segment artificial disc replacement had the potential to restore ROM in the cervical spine. However, additional prospective investigations with longer follow-ups are warranted to gain a deeper understanding of the precise motion characteristics of viscoelastic device implantation.

## 5. Limitations

Although the present study reveals important findings, it is not without limitations. One limitation is the three-year follow-up period, which limits the ability to draw conclusions regarding the long-term performance of viscoelastic devices. Additionally, there may be a risk of selection bias as the study only included patients from a single institution who were treated with a specific type of viscoelastic device by the same neurosurgeon. The heterogeneity in surgical indications among patients may also influence post-operative outcomes.

## Figures and Tables

**Table 1:** Comparison of age, VAS, and angles of ROM by sex at baseline (*n* = 132)

Variables (Mean ± SD)	Female (*n* = 60)	Male (*n* = 72)	*p* Value
Age (yrs.)	60 ± 11.59	56.75 ± 13.13	0.100
≤60, *n* (%)	30 (50%)	46 (63.89%)	0.108
>60, *n* (%)	30 (50%)	26 (36.11%)	
VAS pre-operation	5.23 ± 2.77	4.9 ± 2.66	0.378
ROM (angle)			
C2–C7	40.88± 13.77	39.58 ± 13.09	0.581
FSU	13.4 ± 7.22	11.58 ± 6.31	0.124

Chi-square test and Mann–Whitney U test.

**Table 2:** Comparison of VAS, NDI, and JOA at pre-operation and post-operation stages (*n* = 132).

Variables (Mean ± SD)	Pre-Op	Post-Op	95% CI of the Difference		*p* Value
			Lower	Upper	
VAS	5.05 ± 2.71	1.62 ± 1.16	2.935	3.929	<0.001
NDI	0.47 ± 0.15	0.23 ± 0.17	0.191	0.279	<0.001
JOA	11.33 ± 2.28	15.14 ± 2.14	−4.673	−2.946	<0.001

Pre-op: Pre-operation; Post-op: Post-operation; Paired *t*-test; CI: Confidence Interval.

**Table 3:** Comparison of ROM by time interval and sex within 3–36 months post-operatively (Females = 60, Males = 72).

ROM	Time Interval	Females Mean ± SD	Males Mean ± SD	Time *p* Value	Sex *p* Value	Time × Sex *p* Value
C2–C7	3 mos	55.79 ± 15.51	51.22 ± 13.93	0.001	0.000	0.810
	12 mos	51.08 ± 14.14	44.65 ± 14.17			
	24 mos	49.16 ± 15.07	45.87 ± 13.97			
	36 mos	49.33 ± 14.08	45.63 ± 12.22			
FSU	3 mos	17.43 ± 6.84	14.84 ± 5.44	0.938	0.000	0.929
	12 mos	16.51 ± 6.67	14.98 ± 6.51			
	24 mos	16.72 ± 6.96	14.77 ± 6.25			
	36 mos	16.66 ± 6.82	14.69 ± 6.15			

SD: Standard Deviation, mos: months; General Linear Model (Univariate Analysis of Variance), Time × Sex Group = interaction.

**Table 4:** Comparison of ROM by time and age group within 3–36 months post-operatively (≤60, *n* = 76, >60, *n* = 56).

ROM	Time Interval	Age Group ≤ 60	Mean ± SD > 60	Time *p* Value	Age Group *p* Value	Time × Age *p* Value
C2–C7	3 mos	56.70 ± 14.69	48.67 ± 13.74	0.001	<0.001	0.668
	12 mos	49.87 ± 14.37	44.46 ± 14.12			
	24 mos	50.43 ± 14.12	43.21 ± 14.13			
	36 mos	49.05 ± 14.17	44.96 ± 11.41			
FSU	3 mos	16.20 ± 6.68	15.77 ± 5.60	0.939	0.131	0.894
	12 mos	16.30 ± 6.77	14.83 ± 6.33			
	24 mos	16.13 ± 7.12	15.01 ± 5.90			
	36 mos	15.77 ± 7.28	15.33 ± 5.36			

SD: Standard Deviation, mos: months; General Linear Model (Univariate Analysis of Variance), Time × Age = interaction.

**Table 5:** Comparisons of mean ROM by operated level within 3–36 months post-operatively.

ROM	Operated Level (Mean ± SD) (A)	Operated Level (Mean ± SD) (B)	Mean Difference (A–B)	*p* Value	95% CI	
					Lower	Upper
C2–C7	C5–C6 (52.59 ± 17.02)	C3–C4 (44.70 ± 13.75)	7.88	<0.001	3.29	12.47
		C4–C5 (47.71 ± 9.78)	4.88	0.117	−0.64	10.39
		C6–C7 (47.40 ± 11.70)	5.19	0.003	1.27	9.11
FSU	C5–C6 (18.10 ± 6.43)	C3–C4 (13.97 ± 5.60)	4.13	<0.001	2.11	6.14
		C4–C5 (15.70 ± 4.42)	2.39	0.054	−0.03	4.81
		C6–C7 (13.98 ± 6.79)	4.12	<0.001	2.40	5.84

## Data Availability

The data presented in this study are available upon request from the corresponding author. The data are not publicly available for to the privacy of the patients.
